# Photonic Crystal Biosensor Based on Optical Surface Waves

**DOI:** 10.3390/s130202566

**Published:** 2013-02-19

**Authors:** Valery N. Konopsky, Tanya Karakouz, Elena V. Alieva, Chiara Vicario, Sergey K. Sekatskii, Giovanni Dietler

**Affiliations:** 1 Institute of Spectroscopy, Russian Academy of Sciences, Fizicheskaya, 5, Troitsk, Moscow Region, 142190, Russia; E-Mail: alieva@isan.troitsk.ru; 2 Laboratoire de Physique de la Matiere Vivante, Institut de Physique des Systemes Biologiques, Ecole Polytechnique Federale de Lausanne, CH-1015 Lausanne, Switzerland

**Keywords:** label-free sensing, optical surface waves, critical angle refractometry, biotin-streptavidin binding, PSS/PAH multilayer assembly, kinetics of protein binding

## Abstract

A label-free biosensor device based on registration of photonic crystal surface waves is described. Angular interrogation of the optical surface wave resonance is used to detect changes in the thickness of an adsorbed layer, while an additional simultaneous detection of the critical angle of total internal reflection provides independent data of the liquid refractive index. The abilities of the device are demonstrated by measuring of biotin molecule binding to a streptavidin monolayer, and by measuring association and dissociation kinetics of immunoglobulin G proteins. Additionally, deposition of PSS/PAH polyelectrolytes is recorded *in situ* resulting calculation of PSS and PAH monolayer thicknesses separately.

## 1. Introduction

Photonic crystals (PCs) are materials that possess a periodic modulation of their refraction indices (RIs) on the scale of the wavelength of light [1]. Multiple reflections from the periodic RI boundaries in such materials can lead to the destructive interference of the optical waves and to the formation of bands where light propagation is forbidden. A simple periodic multilayer stack (dielectric mirror) is an example of one-dimensional (1D) PC structure. Such dielectric mirror can reflect all light within its forbidden band gaps, but also can support propagation of optical surface waves along the external border of the mirror. These photonic crystal surface waves (PC SWs) have important features that distinguish them from other waveguide waves and surface waves propagating along the interface under investigation.

The existence of optical SWs in the forbidden band gap of the PC may be deduced from an analogy between electron waves traveling in the periodic potential of the ordinary crystals and optical waves traveling in PCs [2]. In both cases, frequency intervals exist in which wave propagation is forbidden. This analogy may be extended to include surface levels, which can exist in band gaps of electronic crystals (*i.e.*, Tamm states). In PCs, they correspond to optical SWs with dispersion curves located inside the photonic band gap. Sometimes these PC SWs are also called Bloch surface waves [3] or optical Tamm states [4].

Optical surface modes in 1D PCs were studied in the 1970s, both theoretically [5,6] and experimentally [7]. Twenty years later, the excitation of optical SWs in a Kretschmann-like configuration was demonstrated [8,9]. In recent years, PC SWs have been used in ever-widening applications in the field of optical sensors [10–17]. In contrast to surface plasmon-polaritons (SPPs), both *p* -polarized and *s* -polarized optical surface waves can be used in PC SW sensor applications [12]. A direct experimental comparison of the sensitivity of biosensors based either on SPPs or on PC SWs may be found in [18].

In the present article we describe a label-free biosensor device based on PC SWs, where *s* -polarization is used for detection of PC SW, which is sensitive both to adlayer thickness and RI of the liquid, while *p* -polarization is used for detection of the critical angle of total internal reflection (TIR), which is sensitive to RI of the liquid only. The simultaneous registration of these two angles gives possibility to derive both the RI of the liquid and the adlayer thickness. This permits us to segregate the volume and the surface signals from analyte and increase the sensitivity of label-free biomolecule detection.

Label-free optical biosensors play a key role in the selective recognition of target biomolecules and in biomolecular interaction analysis, providing kinetic data about biological binding events in real time without labeling. The advantages of the label-free concept are the elimination of detrimental effects from labels that may interfere with fundamental interactions and the absence of time-consuming pretreatment [19–22]. The disadvantages of all label-free techniques including the most mature one, the surface plasmon resonance (SPR) technique [23], are deficient sensitivity to a specific signal and undesirable susceptibilities to non-specific signals, e.g., to the volume effect of refraction index variations. These variations arise from temperature fluctuations and drifts, and they are the limiting factor for many state-of-the-art optical biosensors. To overcome these disadvantages, we describe the design, realization, and testing of the optical biosensor based on detection of both the PC SW angle and the critical angle.

## 2. Experimental Section

### 2.1. PC SW Biosensor Setup

**Figure 1.**
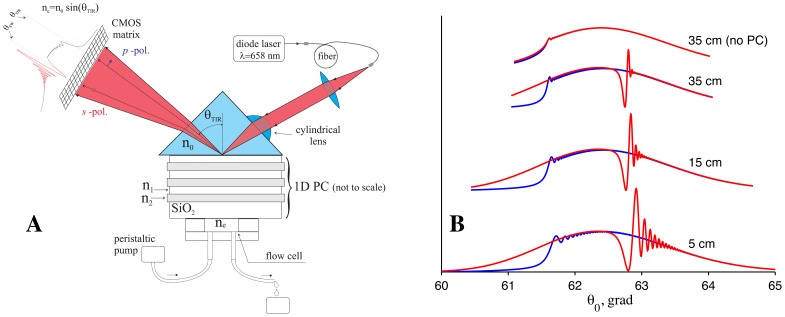
A sketch of the biosensor based on angle interrogation of a PC SW. The typical reflection profile is shown near the CMOS matrix in (**A**) and is illustrated in (**B**) at different distances from the ID PC. The angular resonance curves are shown in red for s -polarization, and in blue for *p* -polarization.

The PC SW biosensor with an independent registration of the critical angle of TIR from the liquid is outlined in Figure 1(A). In this figure, a sketch of the biosensor and typical signals from the photodetector are shown. A laser beam from fiber-coupled diode laser (λ = 658 nm) is sent to the sensor surface through a polarization-maintaining fiber cable (to improve the quality of a beam profile). The beam is focused by a cylindrical lens in such a way that both the excitation angle of one *s* -polarized PC SW (existing in this ID PC) structure and the critical angle of TIR (in *p* -polarization) are contained in the convergence angle of the beam.

Such types of PC SW sensors also possess a spatial selectivity in the direction perpendicular to the plane of Figure 1 (*i.e.*, along the focus line of the cylindrical lens). This fact permits recording of several reactions with an analyte simultaneously if either different ligands are deposited on the PC in several linear target bands or a multichannel fluid cell is used to supply different liquids to the same ligand on the PC surface. In this way, several tests can be performed simultaneously, thus increasing the sensor throughput.

### 2.2. ID PC Structure

The desirable ID PC structure was theoretically deduced using a previously described impedance approach [24]. The following 1D PC structure was designed by this method and was used in experiments: *substrate* /(LH)^3^L'/ *water*, where *L* is a *SiO*_2_ layer with thickness *d*_1_ = 183:2 nm, *H* is a *Ta*_2_*O*_5_ layer with *d*_2_ = 111:2 nm and *L'* is a *SiO*2 layer with *d*_3_ = 341:6 nm. The *SiO*_2_=*Ta*_2_*O*_5_ 7-layers structure (started and finished by *SiO*_2_ layers) is deposited by magnetron sputtering. The prism and the glass plate substrate are made from *BK-7* glass. The RIs of the substrate, *SiO*_2_, *Ta*_2_*O*_5_ and water at λ = 658 nm, are *n*_0_ = 1.514, *n*_1_*= n*_3_*=* 1.47, *n*_2_ = 2.1 and *n_e_* = 1.331, respectively.

### 2.3. Angular Resonance Curves in Reflection Profile of the ID PC Structure

After reflection from the sensor surface, the reflection profile contains information about the TIR angle (transferred by the *p* -polarized part of the beam) and about the angle of the PC SW excitation (transferred by the s -polarized part of the beam). The reflection profile and fringe patterns near the resonance dip and TIR angle are illustrated in Figure 1(B) for different distances from the prism with PC.

The fringe pattern, observed on the larger-angle side of the resonance dip, is the distinguishing feature of all long-range surface waves and a similar fringe pattern was experimentally observed not only with PC SWs [11], but also with ultra long-range SPPs [25–27]. It may be easily inferred that for very large distances, where wave front curvatures of both waves (reflected and reradiated from the surface) equalize, the fringe pattern will disappear, and only the resonance dip will be preserved [25]. Indeed in Figure 1(B) one can see that this fringe pattern is more pronounced at the distance of 5 cm, less pronounced at the distance of 15 cm, and nearly disappears at the distance of 35 cm.

The highest curve in Figure 1(B) is the reflection curve from the prism without multilayer coating (no PC). It is provided to illustrate that the sharpness of the curve near the TIR angle is much higher for our PC structure than the one for the bare surface. Therefore, this setup also may be used as a critical angle refractometer with the enhanced measurement precision, if only *θ*_TIR_ angle is measured.

From Figure 1(B) one can see that the fringe pattern appears not only near the SW resonance (*θ*_PCSW_ = 62.63 grad) but also near the critical angle *θ*_TIR_ = 61.54 grad. This fine interference pattern in the reflection profile of a focused laser beam is the result of enhanced Goos-Hänchen shift [28]. Both interference patterns appearing near the critical angle of TIR and near the resonance dip of long-range surface waves are very important and useful for sensing applications, because the increase in number of points with large values of the first derivative near the dip and near the critical angle leads to an increase in sensitivity to changes in their position. In particular, subpixel registration of a fringe pattern shift becomes possible through detection of lots of these very sharp fringes, which are distributed on many CMOS matrix pixels.

The RI of the liquid may be derived as for classical critical angle Abbe refractometers through the angle of total internal reflection *θ*_TIR_. The liquid RI is then given by

(1) $$n_{e}=n_{0}\sin(\theta_{\text{TIR}}),$$

where *n*_0_ is the RI of the prism in which the critical angle *θ*_TIR_ is measured. To derive changes in the adlayer thickness from changes in the resonance angle Δ*θ*_sw_ and Δ*_ne_* (known from Equation (1)), one can use the dispersion relation valid for both polarizations, which is derived in [24].

### 2.4. Reagents

Hydrochloric acid (37%, HCl, Fluka); ethanol (ACS, Fluka); acetone; ethanolamine (ACS, Fluka); glycine (98%, Aldrich); toluene (99%, Fluka); toluene anhydrous (99.8%, AlfaAesar); 3-triethoxysylilpropylsuccinic anhydride (94%, TESPSA, ABCR); 3-aminopropyl triethoxysilane (APTES, 221.37 Da, Sigma); N-hydroxysuccinimide (98%, NHS, Aldrich); Biotinamidohexanoyl-6-aminohexanoic acid N-hydroxysuccinimide ester (Biotin-X-X-NHS, 567.7 Da, Sigma); N,N-dimethyl-formamide (DMF, Sigma); biotin (Vitamine H, 244 Da, Sigma-Aldrich); streptavidin (*ca.* 60kDa, Sigma); N-(3-dimethylamnopropyl)-N'-ethylcarbodiimide (EDC, Fluka); phosphate buffered saline tablets (PBS, Sigma); polyallylamine hydrochloride (PAH, 58kDa, Sigma-Aldrich); polystyrene sulfonic acid, sodium salt (PSS, 77 kDa, Fluka); sodium chloride (NaCl, Sigma); mouse IgG (Millipore); rabbit IgG (Sigma); goat anti-rabbit (Millipore) and goat anti-mouse IgG (Millipore) were used as received. Citric acid–disodium phosphate buffer solution (CAP, pH = 5.6) was prepared by mixing 42 mL of 0.1 M citric acid (Sigma) with 58 mL of 0.2 M disodium phosphate dodecahydrate (Sigma).

### 2.5. PC SW Substrate Cleaning and Functionalization

Before functionalization, PC SW substrates were sonicated one time in acetone and twice in ethanol (5 min each), each time followed by drying under a stream of nitrogen. After washing, PC SW substrates were treated in plasma cleaner (HARRICK PLASMA PDC-32G) 1 min in medium power, air pressure *ca.* 400 mTorr. Then, these precleaned slides (with expected *OH* bonds on the ultra-hydrophilic *SiO2* surface) were modified to obtain either biotinylated surface for streptavidin/biotin experiments or carboxylate-derivatized surface for experiments with IgG proteins.

In the first case, these precleaned slides were immersed in 1% APTES solution in 95% acetone/water for 5 min to convert the *OH*-terminated *S_i_O*_2_ surface to an *NH*_2_-terminated one. Then, glass slides were dried by nitrogen and desiccated in low vacuum for *ca.* 30 min. To biotinylate the *NH_2_*-terminated surface, the slides were left overnight in solution, where Biotin-X-X-NHS was dissolved by DMF to a final concentration of 500 *μ*g/mL. Afterwards, the slides with a biotinylated surface were sequentially sonicated and thoroughly rinsed with DMF and PBS to remove any excess of Biotin-X-X-NHS.

In the second case, PC SW substrates were modified to obtain carboxylate functionalities [29,30] following further procedure: overnight immersion in a 0.5% (v:v) TESPSA solution in anhydrous toluene in the glove box environment, followed by rinsing and sonication in toluene, acidic water (0.1 M HCl) and drying first under a stream of nitrogen and finally in low vacuum for *ca.* 30 min.

### 2.6. Immobilization of the Streptavidin

**Figure 2.**
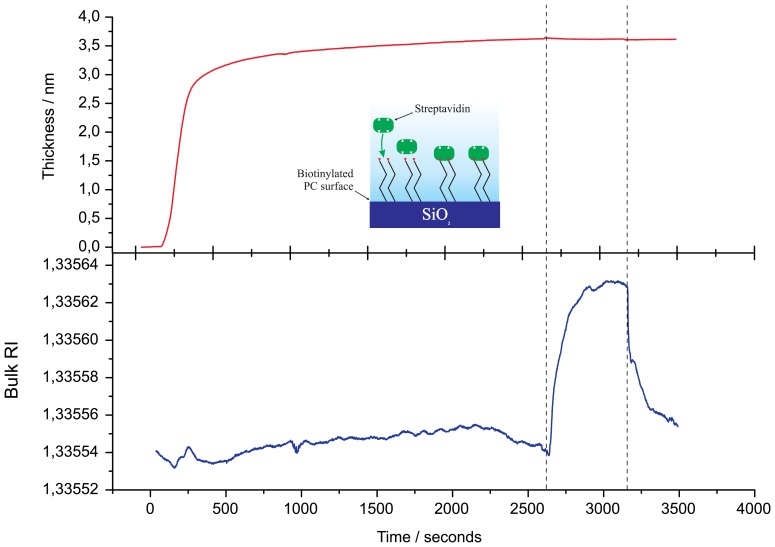
Immobilization of streptavidin on a biotinylated surface.

**Figure 3.**
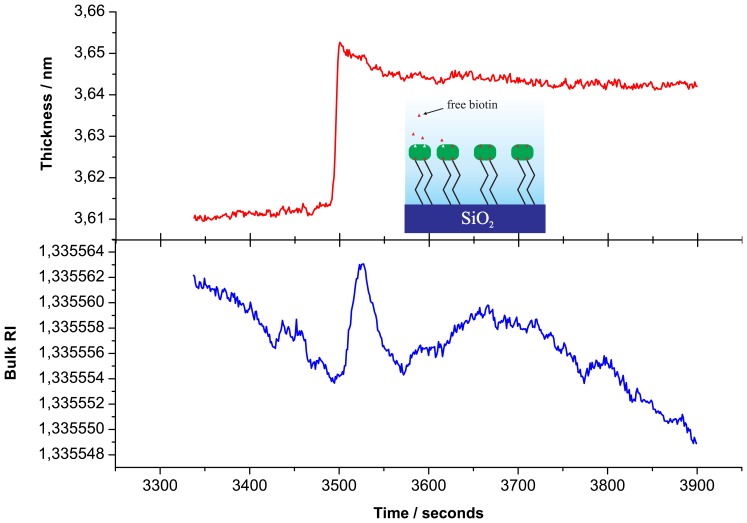
Free biotin binding to the streptavidin monolayer.

Binding of the streptavidin to the biotinylated surface was monitored *in situ* by the PC SW biosensor. Streptavidin (diluted in PBS to a concentration of *c*_s_t_r_ = 12 *μ*g/mL) was run through the flow cell with a volumetric flow rate of *υ*_str_ = 0.3 mL/min. After a streptavidin monolayer formation on the biotinylated surface (see Figure 2), the free biotin was used as a test to detect small molecule binding with the streptavidin monolayer (see Figure 3). Biotin (in a concentration of *c*_biot_ = 0.9 *μ*g/mL) was injected into PBS running through the flow cell with volumetric flow rate of *υ*_biot_ = 0.4 mL/min. All streptavidin / biotin experiments were carried out in PBS (pH = 7.2).

### 2.7. Polyelectrolyte Multilayers

The polyelectrolyte assembly was carried out using the positive polyelectrolyte PAH and the negative polyelectrolyte PSS, 1 mM solutions (concentration calculated with respect to the monomer) in an aqueous solution of 0.1 M NaCl. The PC SW substrate was mounted to the flow cell of the PC SW instrument immediately after plasma treatment and amine functionalization was obtained by flowing 1% (v:v) APTES in water for 5 min to get positively charged surface. Following silanization, the substrate was alternatingly exposed to PSS or PAH solutions for 10 min, starting with PSS. After each adsorption step the substrate was rinsed with water for 100 s, and with an aqueous solution of 0.1 M NaCl for 200 s, and exposed to next electrolyte solution.

### 2.8. Immobilization of the IgG

All further procedures were performed while the carboxylate-derivatized PC SW substrate was mounted to the flow cell of the PC SW instrument allowing *in situ* monitoring of the adsorbed layer thickness and liquid RI. The flow rate during all IgG protein experiments was *ca.* 0.5 mL/min. Bioactivation of the PC SW substrates was obtained by covalent linking of biological ligands using amine coupling via reactive esters [31]. The standard amine coupling includes a three step reaction with EDC/NHS chemistry: activation, ligand linking and deactivation. The activation of the surface was obtained by running 0.1 M EDC/NHS solution in water (freshly prepared) for 5 min following by washing with a buffer (PBS). This procedure was repeated 3 times to increase the activation yield. After the activation, the ligand (rabbit IgG or mouse IgG) was linked to the surface by running a 40 *μ*g/mL solution of it in the CAP buffer for *ca.* 15 min following by washing with the same buffer. After ligand immobilization, the unreacted esters were neutralized by deactivation with 1 M ethanolamine and washed with a coupling buffer for 5 min. Finally the bioactive interface was stabilized by alternate runs of 0.05 M HCl and a coupling buffer for 5 min each, repeated 3 times.

The baseline (the thickness and RI) was registered in the running buffer (PBS) at the beginning of the kinetic measurements. After the baseline stabilization, a PC SW substrate was exposed to a sequence of the receptor concentrations (anti-rabbit or anti-mouse IgG) in the range of 0.5–40 *μ*g/mL in the PBS according to the following cycle: *ca.* 15 min antibody binding followed by 5 min dissociation in the running buffer, 5 min regeneration by 0.05 M HCl mixed with 10 mM glycine and 5 min washing with a running buffer to reset the baseline.

## 3. Results and Discussion

### 3.1. Detection of Free Biotin Binding to the Streptavidin Monolayer

To illustrate sensitivity of the PC SW biosensor device, we present unsmoothed data of free biotin binding on the streptavidin monolayer. Initially, in Figure 2, we present the buildup of the streptavidin monolayer on the biotinylated *SiO*_2_ surface. Then, to remove a streptavidin excess, the flow cell was washed by fresh PBS solution (a region between dashed lines in Figure 2) and biotin was injected into PBS, running through the flow cell (Figure 3).

Figure 3 presents the adlayer thickness changes observed during free biotin (*M*_biot_ ≃ 244 Da) binding to the streptavidin monolayer (top) and the RI changes of the analyte (bottom; during this biotin solution injection. In the color inset the corresponding process is illustrated. It is clear that the biosensor can reliably detect the increase in streptavidin monolayer thickness upon free biotin binding. Figure 3 also illustrates that the change in adlayer thickness occurs with different kinetics from those of the buffer RI. This fact indicates that the volume and surface contributions from an analyte are indeed separated into different registration channels.

### 3.2. Poly electrolyte Multilayers Deposition

**Figure 4.**
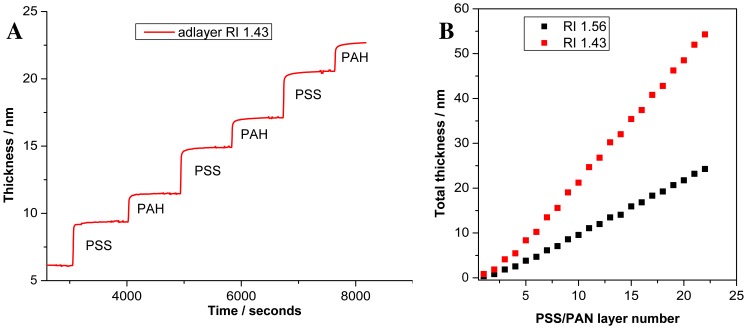
A time slice of the thickness growth of the polyelectrolyte assembly (**A**) and the total layer thickness registered upon polyelectrolyte assembly for different RI of the adsorption layer (**B**).

To demonstrate that response of the PC SW sensor is linear with the thickness of a deposited layer, we record deposition of polyelectrolyte assembly by the device. The 22 polyelectrolyte layers were deposited, started with PSS and ended by PAH. The fragment of *in situ* thickness registration upon polyelectrolyte assembly is presented in Figure 4(A). Figure 4(B) demonstrates increase in polyelectrolyte thickness with growing number of layers. After two initial bilayers, the total adlayer thickness increases linearly with each adsorbed layer and no sign of saturation is observed.

At small adlayer thickness, all optical methods can measure the changes in optical thickness only (*i.e.*, physical thickness of the adlayer multiplied by the adlayer RI: *d*×*n_a_*). Therefore, some assumption about the adlayer RI (immersed in the liquid) is needed (if independent non-optical measurement of *d* is not possible). In our measurement we use *n*_a_ = 1.43 and measure the total thickness of the 22 layers to be equal 54.28 nm. Recalculation of the thickness for other *n*_a_ is also possible. As an example, we recalculate the adlayer thickness, assuming that adlayer RI = 1.56, and receive the total thickness 24.27 nm. This thickness is in good correspondence with results reported in the work [32], where the thickness of one PSS/PAH bilayer was measured to be 2.09 ± 0.03 nm with the assumption that *n*_a_*=* 1.56 (and, thereby, 2.09 × 11 ≃ 23 nm).

To derive the PSS/PAH bilayer thickness more precisely, we should mention that initial PSS and PAH layers have a smaller thickness, probably due to incomplete assembling of the initial monolayers. Taking into account only complete monolayers (*i.e.*, excluding the first two bilayers), we find that PSS monolayer thickness is $d_{\text{PSS}}^{1.56}=1.48\pm 0.04\mathrm{nm}$ and PAH monolayer thickness is $d_{\text{PAH}}^{1.56}=0.98\pm 0.07\mathrm{nm}$, in assumption that *n*_a_ = 1.56, while in assumption that *n*_a_ = 1.43, we get: $d_{\text{PSS}}^{1.46}=3.41\pm 0.07\mathrm{nm}$ and $d_{\text{PAH}}^{1.43}=2.09\pm 0.17\mathrm{nm}$.

That is, for *n*_a_ = 1.43, we measure the PSS /PAH bilayer thickness to be $d_{\text{PASS}+\text{PAH}}^{1.43}≃5.5\pm 0.18\text{nm}$. In the earlier work [33], the thickness of PSS/PAH bilayer was measured to be 5.1 ± 0.2nm according to X-ray reflectometry, and 6.1 ± 0.7 nm (with adlayer RI *n*_a_ = 1.50 ± 0.05), according to ellipsometry. Taken into account that the precise measurement of the adlayer RI for very thin films is hardly possible, it may be stated that our thickness measurements are in a good correspondence with previous ones.

In addition to the previously published results, it is shown that the separate determination of the thickness of each monolayer in the bilayer is possible by our device.

### 3.3. Ligand-Receptor Interactions

**Figure 5.**
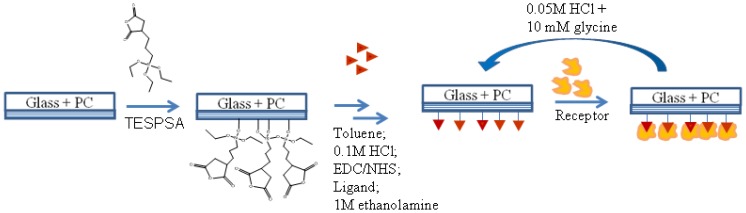
Schematic representation of PC SW surface bioactivation and further receptor recognition.

In this study the applicability of the PC SW optical sensor to characterizing ligand-receptor interactions is demonstrated by measuring association and dissociation kinetics of well-studied systems of immunoglobulin G (IgG) proteins: rabbit and mouse IgG (ligands) and goat anti-rabbit and anti-mouse IgG proteins (receptors). The PC SW substrates were modified to get a biological recognition interface for IgG protein binding, as schematically shown in Figure 5 and described in details in the Experimental Section.

**Figure 6.**
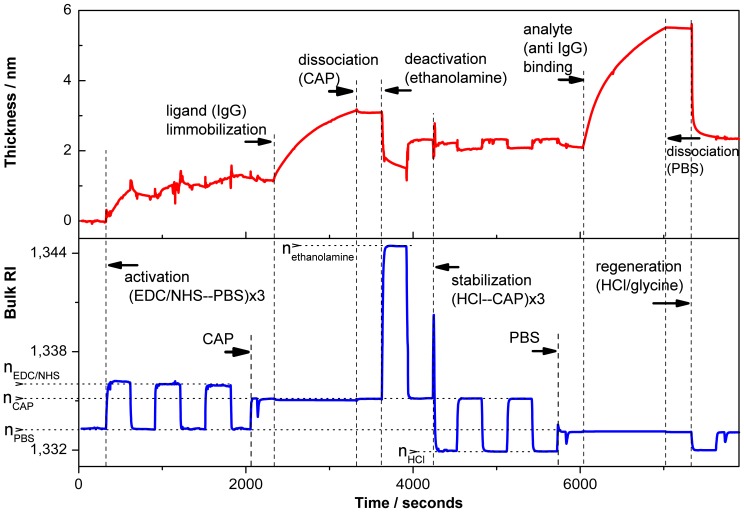
Typical signal obtained by the PC SW optical sensor upon binding of the ligand (40 *μ*g/mL Rabbit IgG) and the analyte (15 *μ*g/mL anti-Rabbit IgG).

In Figure 6, a typical sensogram registered *in situ* by the PC SW optical sensor during ligand immobilization and further kinetic assay is shown (steps are in correspondence with a scheme represented in Figure 5). The changes in adsorbed layer thickness (upper panel) and bulk RI of the medium (bottom panel) are determined simultaneously during the experiment.

In the first step, the free carboxyl groups on the surface were chemically activated using EDC/NHS mixture as described in the Experimental Section. Then the ligand (Rabbit IgG) is immobilized following by ethanolamine deactivation of the remaining activated carboxyl groups and detachment of not covalently bound proteins. The thickness of the adlayer measured by PC SW sensor increases by 2.1 nm after ligand adsorbtion, while after detachment of not covalently bound proteins the thickness of IgG layer decreases to *ca.* 1 nm. Therefore, taking into account that the thickness of IgG monolayer equals 4nm [34], we can estimate the surface coverage of the PC SW substrate with rabbit IgG equals 25% (*i.e.*, one fourth of a monolayer). Finally, a receptor binding to the surface modified with a ligand is observed following IgG complex dissociation in PBS buffer and regeneration procedure. For simple biomolecular interaction analysis, receptors of several concentrations were bound to the immobilized ligand.

**Figure 7.**
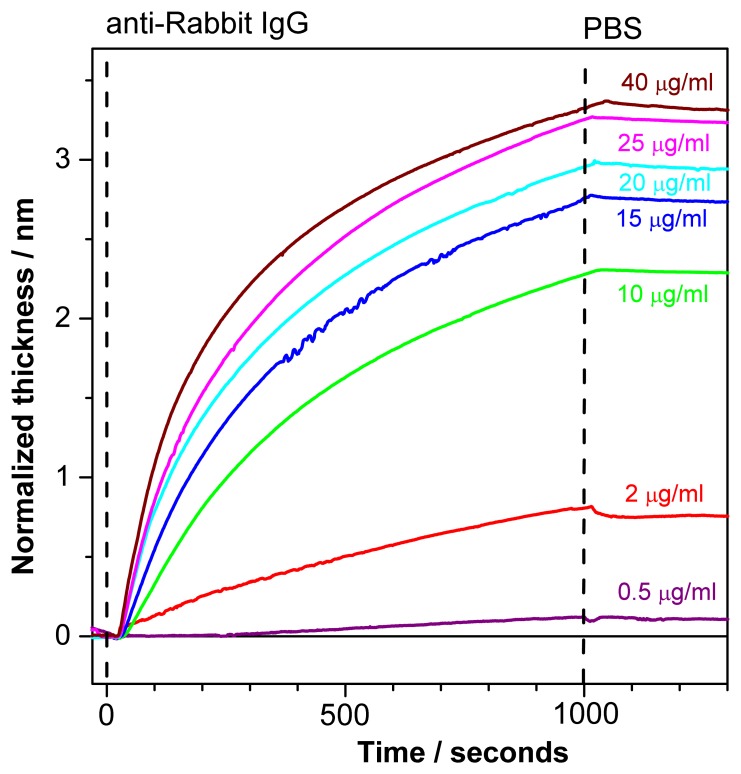
Normalized sensograms of anti-rabbit IgG binding to immobilized rabbit IgG.

Figure 7 depicts normalized sensograms (change in the adlayer thickness as a function of time) for the binding affinity interaction between anti-rabbit IgG at various concentrations and immobilized rabbit IgG. The receptor was injected after baseline stabilization (shown on the graph). The buildup of the signal correlates with rabbit/anti-rabbit IgG complexes formed over time. The PC SW response increases with an increasing antibody concentration (0.5–40 *μ*g/mL). Further the weak dissociation of the complex is seen upon flowing of PBS buffer in the fluid cell.

The response to analyte binding (15*μ*g/mL of anti-Rabbit IgG) in Figure 6 is higher than the corresponding response in Figure 7. This discrepancy could be explained by high reactivity of the ligands (Rabbit IgG) at the beginning of the experiment while no regeneration procedure was applied yet. After the first binding-regeneration cycle, the response to analyte binding becomes stable (not shown here), and only data measured after response stabilization are reliable for kinetics measurements.

## 4. Conclusions

The development of PC-based biochemical sensors is a fast growing area in recent years. The design flexibility of PC structures permits to devise appropriate sensors for any optical wavelength. Excitation of optical surface waves on the PC interface is an effective means to guide and concentrate optical waves in the field of interaction between light and sensing material at the external side of the PC. In the present article we described the label-free biosensor device with 1D PC chips, where the bulk RI of analyte and the adlayer thickness are measured independently. The described here PC SW-based biosensor is commercially available as “EVA 2.0” device [35].

The exploitation of the 1D PCs as substrates, supporting the long-range surface wave propagation, permits researchers to:

1. increase the sensitivity of PC SW biosensors to the level *δd_a_* ≃ 3×10^−13^ m/Hz^1^/^2^ (which corresponds to mass sensitivity *δm_a_* ≃ 0.3pg/mm^2^),
2. segregate surface and volume events in biosensing (which may be an important advantage in applications where temperature and composition of the liquid under study vary over a wide range),
3. enhance the detection of RI variation in Abbe-like refractometer to the level *n_e_* ≃ 10^−7^ RIU/Hz^1^/^2^,
4. use the same PC chip many times, since a thick final *SiO_2_* layer may be effectively cleaned by some active treatment (e.g., in a plasma cleaner).
